# Transcranial random noise stimulation mitigates increased difficulty in an arithmetic learning task

**DOI:** 10.1016/j.neuropsychologia.2015.12.028

**Published:** 2016-01-29

**Authors:** Tudor Popescu, Beatrix Krause, Devin B. Terhune, Olivia Twose, Thomas Page, Glyn Humphreys, Roi Cohen Kadosh

**Affiliations:** Department of Experimental Psychology, University of Oxford, South Parks Road, Oxford OX1 3UD, UK

**Keywords:** Cognitive training, Mental arithmetic, Posterior parietal cortex, Prefrontal cortex, Task difficulty, Transcranial random noise stimulation

## Abstract

Proficiency in arithmetic learning can be achieved by using a multitude of strategies, the most salient of which are procedural learning (applying a certain set of computations) and rote learning (direct retrieval from long-term memory). Here we investigated the effect of transcranial random noise stimulation (tRNS), a non-invasive brain stimulation method previously shown to enhance cognitive training, on both types of learning in a 5-day sham-controlled training study, under two conditions of task difficulty, defined in terms of item repetition. On the basis of previous research implicating the prefrontal and posterior parietal cortex in early and late stages of arithmetic learning, respectively, sham-controlled tRNS was applied to bilateral prefrontal cortex for the first 3 days and to the posterior parietal cortex for the last 2 days of a 5-day training phase. The training involved learning to solve arithmetic problems by applying a calculation algorithm; both trained and untrained problems were used in a brief testing phase at the end of the training phase. Task difficulty was manipulated between subjects by using either a large (“easy” condition) or a small (“difficult” condition) number of repetition of problems during training. Measures of attention and working memory were acquired before and after the training phase. As compared to sham, participants in the tRNS condition displayed faster reaction times and increased learning rate during the training phase; as well as faster reaction times for both trained and untrained (new) problems, which indicated a transfer effect after the end of training. All stimulation effects reached significance only in the “difficult” condition when number of repetition was lower. There were no transfer effects of tRNS on attention or working memory. The results support the view that tRNS can produce specific facilitative effects on numerical cognition – specifically, on arithmetic learning. They also highlight the importance of task difficulty in the neuromodulation of learning, which in the current study due to the manipulation of item repetition might have being mediated by the memory system.

## Highlights

• tRNS was previously shown to enhance cognitive training in an arithmetical task.
• 5-day training on *Calculation* and *Drill* arithmetic was accompanied by tRNS to PFC&PPC.
• Task difficulty was manipulated by including fewer or more problems in training.
• tRNS improved *Calculation* training performance & led to transfer in testing phase.
• tRNS effects were seen only in the difficult task condition.

## 1 Introduction

### 1.1 Types of arithmetic learning

Numerical competence is an increasingly strong predictor of career success and general well-being in today's world (Parsons and Bynner, 2005). Conversely, poor numerical skills have adverse effects on quality of life and world economies (Beddington et al., 2008). Learning arithmetical skills is therefore an important part of an individual's quantitative education, and understanding just how this learning takes place in the brain is of interest not only for basic research but also for understanding conditions such as developmental dyscalculia, where this learning is impaired (Butterworth, 2005, Cohen Kadosh and Walsh, 2007). One of the more salient distinctions among the different types of arithmetic learning is that between learning by *drill* and learning by *calculation* (Delazer et al., 2005, Snowball et al., 2013). The former strategy involves committing arithmetic facts (such as multiplication tables) to long-term memory, whereas the latter involves applying a known algorithm (such as long division) in order to determine the result of a mathematical operation. The two strategies are not mutually exclusive, and are in fact typically used in a complementary fashion when arithmetical procedures are either taught (as part of mathematics education) or applied in everyday calculations. In a previous study that asked participants to perform a mental subtraction task, relative usage of either *drill*- or *calculation*-predominant strategies was predicted based on statistical properties of participants’ RT distributions (LeFevre et al., 2006), specifically on the parameters of the ex-Gaussian function (detailed in Section 2.4.1) that was fit to these distributions.

### 1.2 Neural correlates of arithmetic

At the neural level, drill and calculation strategies appear to be subserved by partly independent mechanisms. For instance, retrieval of memorised arithmetic facts is largely associated with verbal representations in the left angular gyrus (Delazer et al., 2003, Grabner et al., 2009, Ischebeck et al., 2009) – a region believed to more generally encode retrieval of symbolic information (Price and Ansari, 2011). On the other hand, calculation strategies during mental arithmetic have been shown to recruit the bilateral intraparietal sulci (Delazer et al., 2003, Stanescu-Cosson et al., 2000) as well as frontal regions such as the middle and inferior frontal gyri (Delazer et al., 2005, Pesenti et al., 2001). One functional meta-analysis of studies involving both number tasks (e.g. number comparison, counting) and calculation tasks of mental arithmetic (Arsalidou and Taylor, 2011) further clarifies the relative contributions of frontal and parietal cortices in numerical cognition. In particular, accumulating evidence suggests that the former are comparatively more specialised in arithmetical calculation, while the latter (specifically the intraparietal sulcus and the inferior and superior parietal lobules surrounding it) are involved in basic numerical processing. Functional activations of these regions, and in particular of the dorsolateral prefrontal cortex (DLPFC), in tasks involving arithmetical calculations have been found using both magnetic resonance (Cho et al., 2012, Kawashima et al., 2004, Moeller et al., 2015, Rosenberg-Lee et al., 2011, Zamarian et al., 2009) and optical (Near-infrared spectroscopy, NIRS) functional imaging methods (Pfurtscheller et al., 2010, Tanida et al., 2004).

The relative involvement of the regions within this fronto-parietal network in numerical cognition appears to change with increasing expertise with numbers, namely with development and as a consequence of cognitive training. Functional magnetic resonance imaging (fMRI) investigations have shown that, ontogenetically, frontal functions are predominant earlier in development, and are gradually complemented by recruitment of parietal areas such as bilateral intraparietal sulci. This observation holds both for basic processing of symbolic number (Ansari et al., 2005, Holloway and Ansari, 2009) as well as for mental arithmetic (Kucian et al., 2008, Rivera et al., 2005). Cognitively, this suggests decreased reliance on domain-general resources such as attention and working memory during numerical cognition, and, in the case of mental arithmetic, it corresponds to a transfer from methodological (and computationally inefficient) strategies of calculation, to faster and more effortless strategies of memory retrieval (Grabner et al., 2009). Notably, the idea of greater involvement of the DLPFC during the initial stages of skill acquisition and a shift to other brain regions involved in automatic processing, such as the posterior parietal cortex in numerical cognition once skill has been mastered (Cohen Kadosh and Walsh, 2009), is in line with other neurocognitive models of skill acquisition (Chein and Schneider, 2012), including numerical development (Shalev et al., 2007).

### 1.3 Brain stimulation and task difficulty as modulators of arithmetic performance

One means of modulating the cortical changes that accompany arithmetic learning is transcranial random noise stimulation (tRNS). tRNS is a type of transcranial electrical stimulation (tES) whereby a current of random intensity values is delivered, with frequencies distributed across a certain range of the spectrum (Terney et al., 2008). Similar to anodal transcranial direct current stimulation (tDCS), tRNS applied concurrently during a cognitive or motor task has been shown to improve performance, presumably by increasing cortical excitability (Cappelletti et al., 2013, Chaieb et al., 2011, Prichard et al., 2014, Snowball et al., 2013, Terney et al., 2008). There is also preliminary evidence suggesting that tRNS may exhibit a stronger effect than anodal tDCS (Fertonani et al., 2011). In our previous double-blind study, a group receiving tRNS to bilateral DLPFC exhibited a higher learning rate (and transfer to untrained problems) as compared to a sham group (Snowball et al., 2013). These effects did not emerge when we applied the stimulation to the parietal cortex, in a control experiment of the same study. This points to the utility of tRNS for mathematical learning but also suggests that simply stimulating regions known to be involved in the task does not necessarily lead to improved performance.

An important empirical question is not just which *region* to stimulate during cognitive training but also during which *stage* of the cognitive training to deliver the stimulation. Preliminary research provides hints as to the relative stage at which these regions become involved in arithmetical tasks. Ischebeck et al. (2007) examined the learning process involved in a mental arithmetic task with fMRI and found that, as training progressed, learning was accompanied by a relative decrease of activation in frontal areas such as the precentral and inferior frontal gyri, and a relative increase of activation in parietal areas such as the angular gyrus. Similarly, an event-related brain potential (ERP) study of mental arithmetic training (Pauli et al., 1994) found that it is predominantly prefrontal regions that are involved in the first part of the learning (when there is little automaticity), while parietal areas are only involved at later stages, thus imitating arithmetic development. Finally, Delazer et al. (2003) found arithmetic learning – specifically, number matching and fact retrieval – to be accompanied by a shift of activation within the parietal lobe, from the intraparietal sulcus to the left angular gyrus, suggesting a shift in strategy from calculation to retrieval (see also Grabner et al., 2009). Collectively, these previous investigations provide further evidence that cortical recruitment during arithmetic learning shifts from frontal to parietal areas, and suggests that a similar posterior shift of the tES stimulation site may enhance the efficacy of arithmetic training.

A final critical factor that is likely to impact cognitive enhancement is the difficulty of the trained task. Gill et al. (2015) found that anodal tDCS of the left DLPFC applied concurrently to working memory training increased proficiency in a subsequent test, but only when the training programme was sufficiently demanding. Also, task difficulty in two working memory fMRI paradigms was positively correlated with the activation of relevant brain regions, including the left DLPFC (Heinzel et al., 2014, Nagel et al., 2011). The two sets of findings agree with regards to the role of task difficulty, inasmuch as brain stimulation selectively increases excitability in the neural populations most active at the time of stimulation (Silvanto et al., 2008). In numerical cognition, Pope and Miall (2012) found that cathodal tDCS applied to cerebellum was associated with superior performance relative to both sham and anodal tDCS for a difficult serial subtraction task but not an easier serial addition task; and Rütsche et al. (2015) found that, while anodal tDCS improved response latencies in large arithmetic problems, it decreased solution rates in arithmetic problems with smaller operand size. The above studies highlight the importance of task difficulty in the coupling of cognitive training and non-invasive brain stimulation.

### 1.4 Current study

The present study sought to clarify the role of task difficulty as a modulator of performance gain in a tRNS-assisted cognitive training paradigm in the domain of numerical cognition. To this end, we manipulated between groups the difficulty of an arithmetic learning task administered as part of a 5-day training schedule, in a double-blind, sham-controlled design. We chose to manipulate task difficulty by varying the set size, taking ‘easy’ to mean fewer but more frequently-repeated problems, and ‘difficult’ to mean more numerous but less repeated problems. Previous research has indicated that the number of repetitions of an item can affect task difficulty (Warr, 1964). Participants learned drill and calculation arithmetic problems while receiving either tRNS or sham stimulation to bilateral PFC for the first three days and posterior parietal cortex for the remainder of the training. We expected that tRNS would facilitate learning relative to sham and that the magnitude of facilitation would be greater in the more difficult condition.

## 2 Methods

### 2.1 Participants

Thirty-two volunteers (14 female, 18 male; mean age=22.38 years, *SD*=3.37) participated in this study. All participants were right-handed (according to self-report), had normal or corrected-to-normal vision, and met the safety requirements of participation in a tES experiment, i.e. we excluded participants who had a history of neurological conditions (including seizures), implanted metal objects, heart problems or past fainting spells; additionally, all participants were drug- and alcohol-free for the entire week of their participation. Participants were randomly assigned to receive either tRNS or sham stimulation with the constraint that age and gender distributions were similar in the two stimulation groups (tRNS group: 7 females, 9 males; mean age=21.94 years, *SD*=3.55 years; sham group: 7 females, 9 males; mean age=22.81 years, *SD*=3.23 years). The two groups did not differ significantly in terms of age (*F*(1, 30)=0.53, *p>.47*). Informed consent was obtained before the start of each day, and each participant received £50 compensation. Ethical approval was obtained from the Berkshire Research Ethics Committee.

### 2.2 Tasks

#### 2.2.1 Drill and calculation arithmetic

**Fig. 1:**
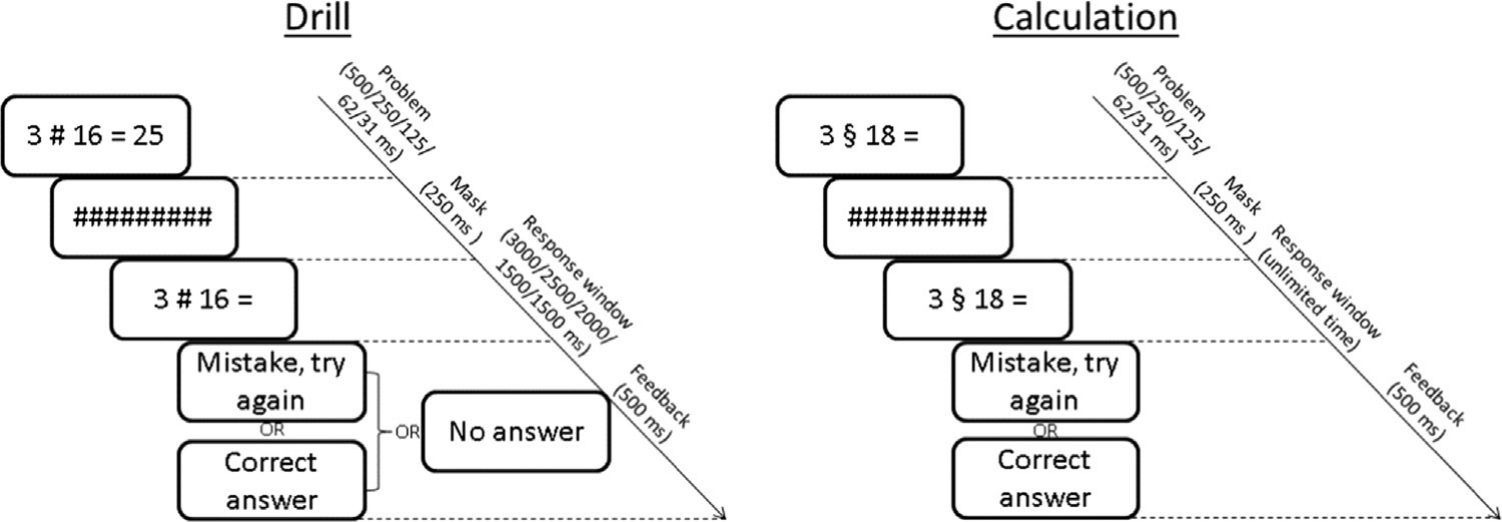
Structure of a trial for each type of problem (left panel: Drill*;* right panel: Calculation).

Drill trials involved remembering associations between pairs of operands and a given result (see Fig. 1, left). Participants were instructed to try to remember these associations throughout the training week. In keeping with the original paradigm (Delazer et al., 2005), the result of each Drill problem was determined using a certain algorithm; participants were not given this algorithm and were instructed to not try and guess it, but instead to learn the associations purely by “drill”. The problem and associated result were displayed on the screen for a duration that halved daily throughout the week (day 1: 500 ms; day 2: 250 ms; day 3: 125 ms; day 4: 62 ms; day 5: 31 ms). A mask was then displayed for 250 ms, followed by a repetition of the problem, this time without the result, and with a limited time window for participants to enter their response (day 1: 3000 ms; day 2: 2500 ms; day 3: 2000 ms; days 4 and 5: 1500 ms). As with previous studies using this paradigm (Delazer et al., 2005, Snowball et al., 2013), the decreasing durations of the first display and response windows aimed to eliminate the effect of day and make the task increasingly challenging as training progressed. In the case of Drill problems, the aim was to encourage participants to produce the result by relying increasingly on memory retrieval and decreasingly on observing the presented result. Feedback on the accuracy of the response was then displayed on the screen for 500 ms, and participants could proceed to the next trial only after giving the correct response.

For Calculation trials, participants were shown a pair of operands and asked to calculate the corresponding result using a given algorithm, which involved multiplication, addition and subtraction (see Fig. 1, right). The two algorithms used were 2×*R*−*L*+1 and 2×*R*+*L*−10 (where *L* and *R* respectively denote the operands to the left and to the right of the “§“ operator). During the initial instructions, participants received their assigned algorithm (see Section 2.3.2 for details) printed on a sheet of paper that they could refer to at any point during the training period. Each set contained 6 pairs of operands. Training material consisted of either one set (“easy”) or two sets (“difficult”), and this difficulty condition was manipulated between-subjects. The terms “easy” and “difficult” do not reflect the difficulty of the problems themselves – which were all matched in difficulty – but of the conditions in which the total amount of training material consisted of these problems repeated more or less frequently (see Section 2.3.2). Participants were instructed to always apply the algorithm rather than make use of any memorised results. Parameters relating to display durations, feedback and proceeding to the next trial were the same as for Drill trials, except that the response time window was not limited.

#### 2.2.2 Control tasks

Participants completed a series of control tasks in order to determine the specificity of tRNS effects. On day 1, prior to the start of training, participants completed the Numerical Operations and Mathematical Reasoning components of the *Wechsler Individual Achievement Test* (WIAT, Wechsler, 1997) in order to have a baseline measure of mathematical ability (relating to arithmetical computations and language-based problems, respectively). In addition, participants completed the (forward and backward) digit-span task and the abbreviated version of the *Attentional Networks Task* (ANT; Fan et al., 2002) before the first and after the fifth training days, in order to examine whether the training phase impacted verbal working memory or attention, respectively. The digit span is a subtest of the WAIS-III (Wechsler, 1997) and requires participants to listen to a string of digits and then reproduce them in the same (forward digit span) or in reverse (backward digit span) order. The ANT produces reaction time measures that can be used to quantify the efficiency of the three networks in the attentional system: alerting, orienting, and executive attention. Briefly, in the ANT, participants respond to the direction (left or right) of an arrow flanked horizontally by two other arrows on either side, with a warning cue appearing either instead or vertically on either side of a fixation cross. The efficiency of the three attentional networks is assessed by measuring how reaction times are influenced by varying the warning signal interval (alerting attention), the validity of the spatial cue (orienting attention), and the contrast between congruent and incongruent flankers (executive attention). The ANT included 3 blocks of 24 trials each.

### 2.3 Procedure

#### 2.3.1 tRNS procedure

All participants received stimulation to the bilateral DLPFC (corresponding to scalp positions F3 and F4 in the international 10/20 EEG system) for the first 3 days of the training phase and to bilateral posterior parietal cortex (P3 and P4) for days 4 and 5. The choice of shifting stimulation site after the temporal midpoint of the training period was informed by previous literature that observed a shift in brain areas involved in arithmetic learning occurring anywhere between the first and the fifth day (Delazer et al., 2005). Current in the form of high frequency noise (100–640 Hz) was delivered by a battery-driven current stimulator (DC Stimulator Plus, Magstim, UK). The current intensity was 1 mA peak-to-peak, with each sample being drawn from a normal distribution with mean 0 μA, and with 99% of all generated amplitude values lying between −500 μA and +500 μA. Stimulation always started at the same time as the onset of the task. The stimulation duration was set to 20 min for the tRNS group and to 30 s for the sham group, both flanked by a 15 s upward and downward ramp. The current was delivered through two 4×4 cm electrodes that were secured in place using an elastic strap and placed in saline-soaked sponges to improve electrical contact with the scalp and reduce the risk of skin irritation. Neither the participant nor the experimenter was aware of the type of stimulation received. At the end of the experiment all participants were asked whether, judging by the sensation that they felt underneath the electrodes during the stimulation, they thought they were in the tRNS or in the sham stimulation group. This was done in order to verify the stimulation condition blindness. Previous studies suggest that tRNS with the current intensity used here is not perceivable (e.g., Ambrus et al., 2010; Fertonani et al., 2015).

#### 2.3.2 Training phase

In a similar experimental design to Delazer et al. (2005) participants completed a five-day training phase, followed – on the final day – by a testing phase. The training involved daily sessions during which participants performed two types of arithmetic learning tasks: Calculation, where two numerical operands were connected by the operator §, and Drill, where the operands were separated by the operator # (see Fig. 1 in Section 2.2.1). The result was always a double-digit number, which the participants were asked to enter using a standard computer keyboard. Both conditions were presented in blocks of 18 trials. Within each day, there was an equal number of Calculation and Drill blocks, and their order was alternated. In keeping with the original paradigm (Delazer et al., 2005), and based on the observation that RTs speed up on subsequent days, the total number of blocks was different for each day (10 blocks on day 1, 12 on day 2, 14 on day 3, 16 on day 4 and 14 on day 5), in an attempt to have daily training sessions of equal duration. In order to manipulate task difficulty between subjects, only one set of operand pairs was used for the training phase in half of the sample (the “easy” condition), meaning there were 6 Calculation problems and 6 Drill problems in total, whereas for the other half (the “difficult” condition), two sets of operands were used (i.e., 12 possible problems for each of the two types of learning). In the easy condition there were two repetitions of each problem per block. In the difficult condition, where the number of individual problems doubled, the number of repetitions per block correspondingly decreased to one. Within each stimulation condition, participants were randomly assigned to one of four groups defined by the existence of 2 algorithms for the Calculation problems and 2 different sets of numerical operands used in all problems. Groups 1&3 and 2&4 were given the algorithms 2×*R*−*L*+1 and 2×R+*L*−10, respectively for calculation; and different sets of operands were used for groups 1&2 and 3&4, respectively. This assignment was done for the purpose of balancing the parameters of the task for each group, and was independent of the assignment of participants to either the sham or the tRNS group. Both the training and the testing phase tasks were implemented using E-Prime (PST Inc., Pittsburgh, USA) on a desktop PC. Reaction time (RT) – measured from the onset of the problem display and until the final (second) digit of the response was pressed – and accuracy were recorded for each response. For all tasks, participants were seated in front of a 19” monitor, at a distance of approximately 60 cm.

#### 2.3.3 Testing phase

Following the end of the training phase, on day 5, participants underwent a single-session testing phase that aimed to assess whether the skills acquired for the two types of problems during training would transfer to new problems (pairs of operands). This session had the same structure as the daily training sessions, except (i) the operands included both “old” pairs from the training phase and “new” untrained pairs; (ii) feedback was not given and participants continued to the next trial regardless of the accuracy of their response. In keeping with the original paradigm (Delazer et al., 2005), in the easy condition, three sets were used for the testing session: one set of old problems and two sets of new problems. For the difficult condition, due to an error in the programming of the task script, the testing session used only two sets: one set of old problems and one set of new problems. A total of 96 trials were presented for each problem type and in each difficulty condition, with 50% of trials comprising old problems. Participants were asked to provide their best guess for “new” Drill problems (whose results they had not learned) in order to check whether the underlying algorithm had been deduced.

### 2.4 Analyses

A number of participants had inordinately low accuracy scores for old Drill problems in the testing phase. In contrast to the training phase in this phase the problems are presented without the solution. This result indicates that those participants had not learned the associations between operand pairs and their corresponding result in the training phase and instead just transcribed the isolated result. To assess whether those participants just guessing the answer at this stage we considered their performance at the chance level if their accuracy was ±2.5 *SD*s from the mean accuracy of new Drill problems (which cannot be solved correctly as they were not learned during the training). This resulted in the 0.50 mean accuracy value as a cut-off for defining outliers for old Drill problems and led to the exclusion of 11 participants' data (34%; 4 from tRNS, 7 from sham; 3 from “easy”, 8 from “difficult”) from all analyses of Drill problems, for both training and testing. Such a restricted remaining sample precludes interpretation of the group data (see Section 4) and we report the Drill results for the sake of transparency.

RT outliers (*Mean*±2.5 *SD*s, comprising <1% of trials) were removed separately for each type of problem and for each participant. Mean accuracy and mean RTs on correct trials for Drill and Calculation problems were submitted to separate 4-way mixed model analyses of variance with Stimulation Group (tRNS vs sham) and Difficulty (easy vs difficult) as between-subjects factors, and Day (1 through 5) and Block (1 through 5) as within-subjects factors. For each problem type, only the first 5 blocks of each day were taken into consideration to allow factorial analyses (Snowball et al., 2013). The Greenhouse-Geisser correction was applied in cases where data violated the sphericity assumption. Learning rate was estimated using the power law of practice (Newell and Rosenbloom, 1981, Rickard, 1997; see Supplementary Materials for details).

#### 2.4.1 RT distribution analysis

Analyses of RT distributions can often provide more detailed descriptions than just standard measures of central tendency, which can obscure finer aspects of performance. In some cases the shapes of these distributions can be used to make inferences about underlying processes, allowing for the testing of hypotheses that are indistinguishable when only comparing mean RTs (for a review, see Balota and Yap, 2011). The *ex-Gaussian* function provides good fits for empirically obtained RT distributions, with parameters that are intuitively interpretable on the distribution histogram. This function is defined as the convolution of a normal and an exponential distribution (Heathcote, 1996, Heathcote et al., 1991, Lacouture and Cousineau, 2008). Its right-hand tail is skewed due to the exponential, and this makes it a good fit to RT distributions, which are often positively-skewed, with most RTs clustering at the faster end of the scale. Fitting the ex-Gaussian to RT data provides, for each participant, an estimate of *µ* and *σ* (the mean and standard deviation of the normal component) and of *τ* (the mean of the exponential component, i.e. the positive skew); the mean RT is approximated by the sum of *µ* and *τ*. *µ* reflects the position of the distribution along the *x*-axis, and so an increase in *µ* reflects a uniform rightward shift of the main body of the distribution. An increase in τ corresponds to a rightward skew of the distribution, which can reflect a slowing on some trials due to “later” cognitive processes than those contributing to *µ* (e.g. response selection). Finally, *σ* indexes the RT variability in the normal component of the distribution. For tasks relying on working memory, an increase in *µ* has been suggested to reflect a global slowing of memory retrieval, with reliance on reasoning contributing predominantly to *τ* (Schmiedek et al., 2007). In numerical cognition, *µ* and *τ* have been used to identify patterns of individual differences in simple mental arithmetic strategies (LeFevre et al., 2006).

Here, for each training day of each participant, the RT distribution for each type of problem was submitted to an analysis which estimated the ex-Gaussian parameters *μ*, *σ* and *τ* (Lacouture and Cousineau, 2008). The RT distributions were calculated at day level and not at block level because at least 100 observations per distribution are typically needed to adequately fit the ex-Gaussian function (Ratcliff, 1979).

## 3 Results

Participants were unable to correctly identify their stimulation group (see Table S3 in Supplementary Materials for details).

### 3.1 Control tasks

ANT (alerting, orienting, and executive attention) and working memory scores were submitted to Stimulation Group×Day ANOVAs. There were no significant effects for either (all *ps*>.15; see Table S4 in Supplementary Materials for details). Critically, the groups did not differ in baseline mathematical ability (all *ps*>.64).

### 3.2 Training phase

#### 3.2.1 Response accuracy and latency

**Fig. 2:**
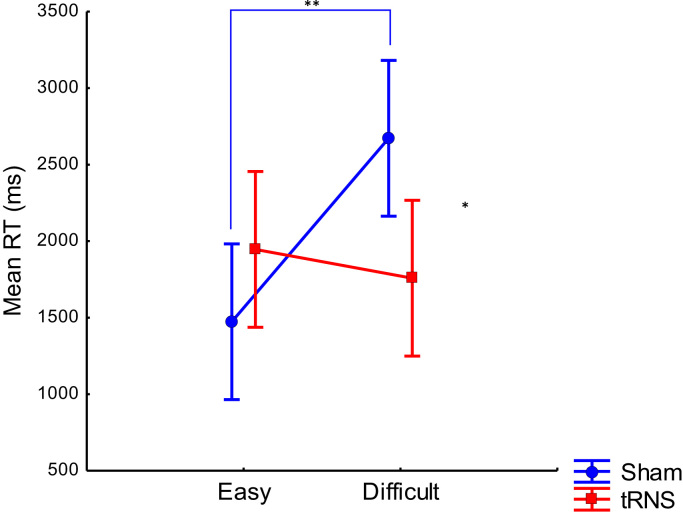
RTs on Calculation trials, as a function of Difficulty and Stimulation Group. Data is collapsed across all five training sessions. Error bars represent 95% confidence intervals. * *p*<.05, ** *p*<.01.

There were no significant effects for Drill problems in terms of accuracy or RT, and no significant effects for Calculation accuracy (all *ps*>.3). The only significant effect was for Calculation RTs, a Stimulation Group×Difficulty interaction (*F*(1, 28)=7.79, *p*<.01, η_p_^2^=.22), depicted in Fig. 2; all other effects were non-significant (all *ps*>.18). Planned comparisons revealed that the tRNS group was significantly faster than the sham group in the difficult condition (*F*(1, 28)=6.78, *p*<.05, *d*=1.39), but not in the easy condition. In addition, for the sham group only, the difficult condition was slower than the easy condition (*F*(1, 28)=11.64, *p*<.005, *d*=1.82); this effect within the sham group was confirmed by a decreased learning rate in the difficult as compared to the easy condition (see Supplementary Materials and Fig. S1 for details).

#### 3.2.2 RT distribution analysis

**Fig. 3:**
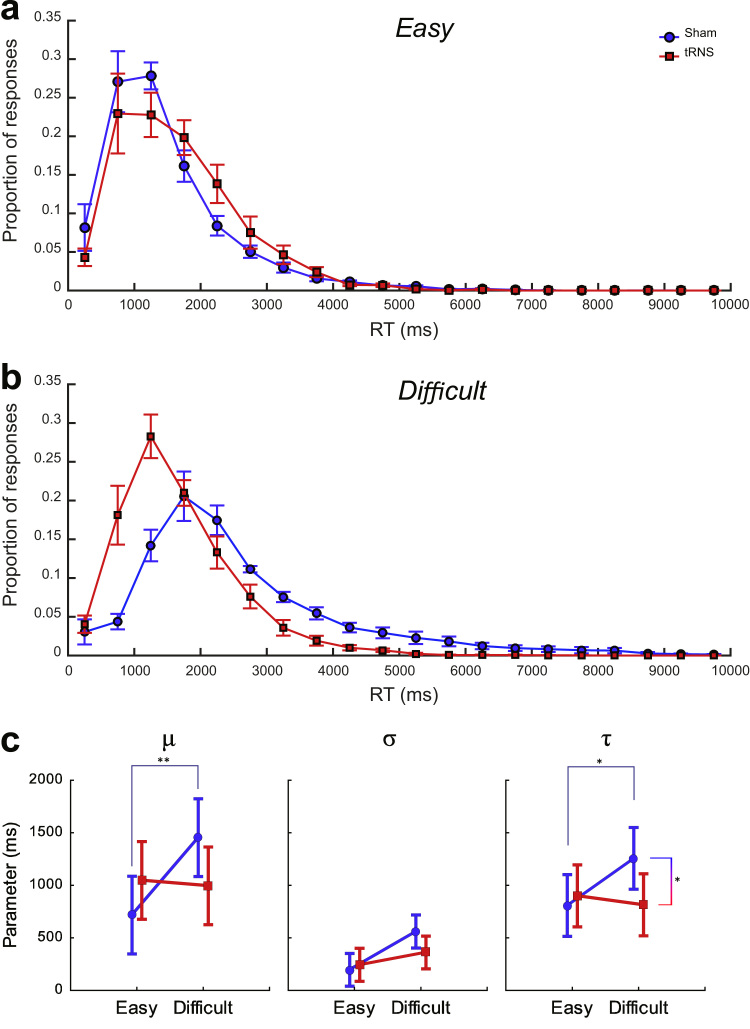
Calculation RT distributions for the (a) easy and (b) difficult conditions as a function of Stimulation Group. Data points represent the mean proportion of responses across participants, within 500 ms-wide bins. (**c**) The ex-Gaussian parameters – *µ* (mu), *σ* (sigma) and *τ* (tau) – that describe these distributions as a function of Difficulty and Stimulation Group. Error bars represent 95% confidence intervals. * *p*<.05, ** *p*<.01.

Fig. 3a and b shows the RT distributions for Calculation problems, for each difficulty condition. Whereas the distributions largely overlap in the easy condition, in the difficult condition the RT distribution peaks earlier in the tRNS than in the sham group; this is indexed by the smaller μ, while the shallower exponential tail is indexed by the smaller τ (see below). Separate Stimulation Group×Difficulty×Day mixed-model ANOVAs were conducted on each ex-Gaussian parameter. In line with the RTs results, the only significant effects were for Calculation: a Stimulation Group×Difficulty interaction on μ (*F*(1, 28)=4.76, *p*<.05, η_p_^2^=.15) and a trend towards the same effect on τ (*F*(1, 28)=3.45, *p*=.074, η_p_^2^=.11) (see Fig. 3a); all other effects were non-significant (all *p*s>.33). Planned comparisons revealed that both μ and τ increased with difficulty in the sham group (*μ*: (*F*(1,28)=8.33, *p*<.01, *d*=1.54; *τ*: *F*(1, 28)=4.88, *p*<.05, *d*=1.81), but not in the tRNS group (all *ps*>.68). Additionally, the sham group had higher τ values than the tRNS group in the difficult (*F*(1, 28)=4.73, *p*<.05, *d*=1.16), but not in the easy condition (*p*>.65); the same contrast approached significance for *μ* (*F*(1, 28)=3.32, *p*=.083, *d*=0.97). This indicates that, in conditions of increased task difficulty, tRNS reduced both the mean and the tail of the RT distribution.

### 3.3 Testing phase

**Fig. 4:**
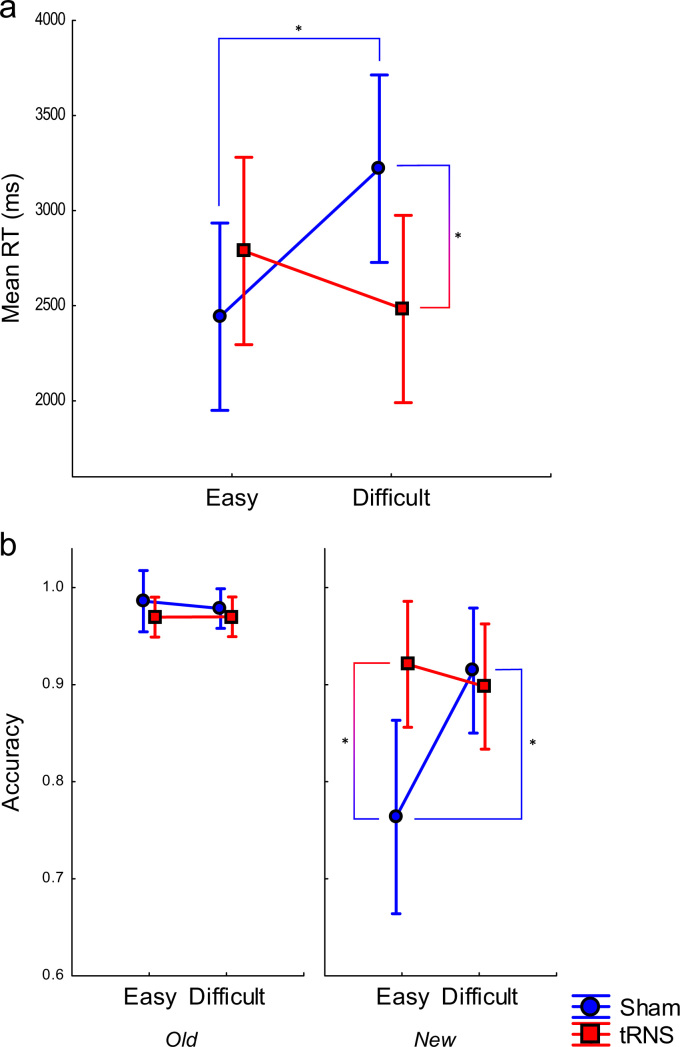
(a) RTs and (b) accuracy on Calculation problems in the testing phase, as a function of (a) Difficulty and Stimulation Group or (b) Difficulty, Stimulation Group and Problem Novelty. Error bars represent 95% confidence intervals. * *p*<.05.

RT and accuracy for each problem type were submitted to Stimulation Group×Difficulty×Problem Novelty (old vs new) mixed-model ANOVAs. Numerical Operations was included as a covariate for Calculation analyses, to partial out the influence of individual differences in baseline numerical ability when comparing the skill transfer from trained to untrained material. There were no significant effects for Drill problems (all *ps*>.63). All effects in the RT analyses of Calculation problems were non-significant (all *ps*>.42) except a Stimulation Group×Difficulty interaction (*F*(1, 28)=5.07, *p*<.05, η_p_^2^=.17), depicted in Fig. 4a. It is noteworthy that this effect is similar to the one obtained for training Calculation RTs (see Fig. 2). Subsidiary analyses revealed lower RTs in the tRNS than in the sham group within the difficult (*F*(1, 28)=4.70, *p*<.05, *d*=1.16) but not in the easy condition (*p*>.32), and also higher RTs as difficulty increased in the sham group (*F*(1, 28)=5.23, *p*<.05, *d*=1.22) but not in the tRNS group (*p*>.37). These effects were independent of novelty (Stimulation Group×Difficulty×Problem Novelty: *p*>.74).

For Calculation accuracy, all effects were non-significant (*p*>.11), except a Stimulation Group×Difficulty×Problem novelty interaction (*F*(1, 28)=5.30, *p*<.05, η_p_^2^=.18) (see Fig. 4b). The Stimulation Group×Difficulty interaction was significant for new (F(1, 28)=5.73, *p*<.05, η_p_^2^=.20) but not for old problems (*p*>.4). Subsidiary analyses revealed lower accuracy in the sham than in the tRNS group, in the easy condition for new (*F*(1,28)=7.45, *p*<.05, *d*=1.46) but not for old problems (*p*>.24*)*, suggesting that the increased effort presented by new (untrained) problems was reflected in a decreased mean accuracy only for sham but not for tRNS. Subsidiary analyses for new problems in the sham group also revealed increased accuracy as difficulty increased (*F*(1,28)=6.89, *p*<.05 *d*=1.40). This suggests that, for the participants in the sham group, performance with untrained problems was worse when the training was less effortful.

## 4 Discussion

We found that tRNS improved performance on arithmetic problems requiring the application of a formula to a set of operands (“Calculation”). This improvement was reflected in a stabilisation of performance and learning rate even as difficulty increased, and also generalised to untrained problems. Cumulatively, these results suggest that tRNS-assisted training mitigates the effect of increased difficulty.

### 4.1 Mitigation of increased difficulty during training phase

Stimulation group differences were only observed when the task was more difficult. Additionally, only the sham group incurred the performance cost for increased difficulty. These results are consistent with previous findings that the effectiveness of tDCS in mental arithmetic (Rütsche et al., 2015) and working memory tasks (Gill et al., 2015, Pope and Miall, 2012) increases with task difficulty. They are also consistent with the long-standing proposition in the learning and memory literature that during a learning task, a minimum level of *desirable difficulty* optimises long-term outcomes by promoting transfer of knowledge (Bjork, 1994).

While we term the manipulation of set size in the current study as difficulty manipulation, one might suggest that the manipulation of the number of repetitions caused the performance differences rather than difficulty manipulation. However, previous research in cognitive psychology have indicated that performance differences as indicated by reaction time and/or accuracy are an indicator of task difficulty, and task difficulty can be created at different levels such as the perceptual, memory, response, or cognitive level. Our definition of difficulty manipulation in the current context is supported by the performance differences between the groups, which was significant when performing the task without brain stimulation (sham group). Indeed, for sham stimulation the differences in reaction time as a function of set size is an indicator of task difficulty. Our RT distribution analysis also indicates that the effect is not likely to be due to perceptual differences, but rather due to a later level of information processing. The effect of tRNS as a function of difficulty might have being mediated in the current study by the memory system. Previous tRNS studies, including the current one, have indicated the effect of tRNS on learning and memory (Cappelletti et al., 2013, Fertonani et al., 2011, Snowball et al., 2013). In this case tRNS, when delivered under a condition of low number of repetitions (the “difficult” condition), might have yielded greater benefit than sham stimulation by improving procedural memory. For participants trained in the easy condition (higher number of repetitions), the beneficial effect of the tRNS might not have been fully “exploited”, possibly due to a ceiling effect. Alternatively, due to the small number of Calculation problems in the easy condition, participants might have simply switched to rote retrieval, a strategy that might not have benefited from tRNS in the current study.

The group difference effect in mean Calculation RTs in the difficult condition is complemented by the effect observed for the parameters of the ex-Gaussian fit to the RT distribution; namely, tRNS decreased the normal (μ) as well as the exponential (τ) component as compared to sham. While the effect for *μ* can be viewed in direct relation to the decreased mean RTs observed in the ANOVA, the interpretation of the effect for *τ* is more subtle. Greater values of τ imply a stronger rightward skewing of the RT distribution, which in turn has been suggested to underlie later cognitive processes (Luce, 1986), as opposed to earlier or more perceptual ones such as the time required to register the problem and physically make a response. In a study where participants solved addition problems in different formats (Arabic numbers or number words) and reported their strategy (calculation or memory retrieval) at each trial, an ex-Gaussian analysis suggested that, for each format, retrieval trials contributed predominantly to *μ* and calculation trials to *τ* (Campbell and Penner-Wilger, 2006). Similarly, LeFevre et al. (2006) found that *τ* discriminated well between the different self-reported strategies (such as retrieval, transformations or counting) used in a simple subtraction task, having small values for retrieval and large values for counting. Applying such an interpretation – based on the assumption that RTs on calculation trials will be sampled from a different distribution than RTs on retrieval trials – to the present study, the smaller τ of the tRNS group might reflect faster or more efficient calculation. This, in turn, might reflect – and be a result of – an improvement of working memory capacity, a hypothesis supported by previous work associating low τ values in choice reaction tasks with better working memory abilities (Cowan and Saults, 2013, McVay and Kane, 2012, Schmiedek et al., 2007, Shahar and Meiran, 2015) or, alternatively, to lower working memory demands related to experimentally induced working memory load (Shahar et al., 2014).

During training, the effect of stimulation did not vary according to day. This may be due to different reasons. First, the interaction with the day factor might have been masked by the confounding effect of problem presentation time, which decreased with each session, thus making the task more difficult as training progressed. Second, it might be that tRNS – in contrast to, for instance, tDCS – is effective from the very beginning of the stimulation, with its immediate effect perhaps due to the oscillatory nature of its current. This would conceal the facilitative effect within a smaller time scale than would be detectable with the present analyses, and would instead give rise to the relatively constant difference in RTs between the groups observed when viewed at the day level. Third, it is possible that the group difference found in the difficult condition was due a pre-existing baseline difference between the groups. However, the two last explanations are unlikely, due to several reasons: (i) the participants were randomly assigned to stimulation groups; (ii) the groups did not differ in terms of the baseline of calculation ability (as measured before the start of stimulation by the Numerical Operations component of the WIAT); and (iii) it was the learning rate (*α*), rather than the initial performance (*B*), that was higher for the tRNS group in comparison to sham (see Supplementary Materials for details).

### 4.2 Transfer to untrained problems during testing phase

During Calculation testing, accuracy for new problems was lower in the easy than in the difficult condition, for the sham group. A likely explanation is that, in the easy condition training consisted of relatively few Calculation problems, these problems were being solved by memory recall in this group as opposed to actual calculation. This, in turn, made new problems presented in the testing phase more difficult, since at this point mental calculation, which was not trained to the same extent as in the difficult condition, now had to be used. This effect did not occur for the tRNS group, which also showed better performance than sham in the easy condition. This might suggest that here, perhaps due to a facilitation of calculation strategies during training (Snowball et al., 2013), such a strategy was used more consistently during both training and testing.

Problem novelty did not interact with stimulation group and task difficulty for Calculation RTs. This effect suggests that the tRNS facilitation effect was transferred from the trained to the untrained material. This is similar to the results of Snowball et al. (2013), where transfer of RT facilitation was also found to occur across old and new Calculation problems. The finding of a transfer effect in the current study is in line with previous tRNS studies that showed short (Cappelletti et al., 2013) or long-term transfer effect (Snowball et al., 2013), and therefore highlights the potential of tRNS as a tool to increase transfer effects. However, the longevity of the tRNS effects in the current study is unknown.

A large proportion of participants had to be excluded from the Drill analyses due to their evidently incorrect strategy for performing the task. We have reported the Drill results for the sake of transparency, however the remaining sample size is unfortunately too small to allow reliable group inference, and our null results for Drill trials – in both the training and the testing phase – should be seen in this light and interpreted with caution.

It should be noted that the facilitative effects presented here were obtained using a novel tRNS montage that implied shifting the stimulation site mid-training. This aimed to reproduce the putative frontal-to-parietal shift that accompanies arithmetic learning (Delazer et al., 2003, Ischebeck et al., 2007, Pauli et al., 1994). Mental arithmetic consists of complex operations carried out across a network of predominantly parietal and frontal regions. While both are integral to a person's ability to carry out these operations, frontal areas are comparatively more engaged during calculation while parietal areas appear involved in basic numerical processing more generally (Arsalidou and Taylor, 2011). Importantly, as noted above, these respective roles of the frontal and parietal cortices in performing mental operations shift dynamically with increasing expertise (e.g., Rivera et al., 2005). Our intention here of reproducing said frontal-to-parietal shift was done, in turn, in an attempt to have the relevant region stimulated at the most relevant time, and thus enhance the amount of facilitation. However, the current dataset does not allow a direct comparison in that sense with the results of previous studies that have used fixed stimulation site montages with numerical cognition training tasks (Cappelletti et al., 2013, Snowball et al., 2013). It is hoped that future studies will investigate the relative efficacy of the fixed and shifting stimulation site paradigms, for instance by manipulating the time point during training when the stimulation shift takes place. This will allow to ascertain which optimal montage should be applied to hypothesised brain regions, in order to enhance the efficacy of tRNS-assisted training in any domain of cognition. Also, the present data set, while including a sham tRNS control, does not benefit from a control stimulation site to enable it to verify the specificity of the found effects to the particular stimulation montage employed here. Using a similar training paradigm as in the present study, Snowball et al. (2013) have suggested that the positive effect of tRNS on Calculation learning and transfer was specific to the prefrontal stimulation site but not also to a posterior parietal control site. However, a more complete characterisation of the specificity of a particular stimulation montage to arithmetic learning can be achieved by future studies that may choose to employ further control sites based on brain areas likely to have little specific relevance for numerical cognition.

Finally, a potential concern might be the uneven distribution of new and old problems between the two difficulty conditions, namely that (fewer) new problems are repeated more often in the difficult than in the easy condition. However, the main conclusions of this study are based on the comparison between sham and tRNS in each difficulty condition, and therefore this potential problem does not impact the interpretation of the main results.

### 4.3 tRNS selectivity

Stimulation did not affect performance on control (attention and working memory) tasks, suggesting that, as in some tES studies of numerical training (Cappelletti et al., 2013, Cohen Kadosh et al., 2010, Snowball et al., 2013), the effects were specific to the trained material and do not extend to domain-general abilities. It is, however, very difficult to rule out the possibility that some hidden – beneficial or impairing – effects exist for cognitive processes other than the ones tested (Brem et al., 2014, Iuculano and Cohen Kadosh, 2013, Sarkar et al., 2014).

### 4.4 Conclusions

Overall, the results of this study corroborate the ability of tRNS to selectively improve cognitive training outcomes – including transfer effects – in high-level cognitive training as a function of task difficulty. These effects render tRNS a promising tool to improve cognitive training outcomes. Future studies should shed light on whether tRNS can be particularly helpful when the level of difficulty is subjectively or objectively high, such as in specific learning disabilities, or in cognitive decline due to ageing. At the same time its exact effect at the cognitive (e.g., memory systems) and neural level (e.g., the effect on brain networks, neurochemicals) should be further examined to increase our understanding and exerts it effect to a greater extent.
