# Supplementary material for: Transcranial random noise stimulation mitigates increased difficulty in an arithmetic learning task
Source: Neuropsychologia. 2016 Jan 29;81:255–64. doi: 10.1016/j.neuropsychologia.2015.12.028 (PMC4749538; doi:10.1016/j.neuropsychologia.2015.12.028)
Supplement: Supplementary file 1 — Supplementary material [file mmc1.docx]

# Supplementary Methods

## Procedure

Table S1: Number of problems and of repetitions during the training phase. All parameters are equal across the Calculation and Drill tasks.

|  | Easy | Difficult |
| --- | --- | --- |
| Number of individual problems | 6 | 12 |
| Number of repetitions per problem per block | 2 | 1 |

Table S2: Number of problems and of repetitions during the testing phase. All parameters are equal across the Calculation and Drill tasks.

|  | Easy | | Difficult | |
| --- | --- | --- | --- | --- |
|  | new | old | new | old |
| Number of individual problems | 12 | 6 | 6 | 6 |
| Number of repetitions per problem per block | 1 | 2 | 2 | 2 |

## Modelling of Calculation learning curves

Curves described by power laws have been shown to be a good model for the improvement in RTs that accompanies practice-based learning in several types of cognitive domains, including mental arithmetic and memory recall of arithmetic facts (Newell & Rosenbloom, 1981; Rickard, 1997). Using a power law, RT is expressed as a function of time (day number) as follows:

RT = B × N^-α^,

where *N* is day number, *B* is a constant signifying initial performance (RT) for N=1, and *α* is the learning rate; a positive *α* is indicative of decreasing RTs, with higher values reflecting faster learning. We fitted this power law to Calculation RTs and looked for an effect of stimulation group on *α* (Iuculano & Cohen Kadosh, 2013; Snowball et al., 2013). We used non-linear estimation, using the least squares method, to estimate the unknown parameters *B* and *α* for each participant, across the five sessions.

# Supplementary Results

## Stimulation perception

Table S3 shows the participants' reports of their perceived stimulation condition. Participants were unable to correctly identify their stimulation group (Yates corrected χ^2^=.75, *p>.1*). This finding is in line with previous studies suggesting that tRNS offers good blinding control (Ambrus, Paulus, & Antal, 2010; Fertonani, Pirulli, & Miniussi, 2011; Prichard, Weiller, Fritsch, & Reis, 2014).

Table S3: Contingency table with participants' perceptions of stimulation condition.

| Group | Response: "tRNS" | Response: "I do not know" | Response: "Sham" |
| --- | --- | --- | --- |
| *tRNS* | 2 | 6 | 8 |
| *Sham* | 6 | 3 | 7 |
| *TOTAL* | 8 | 9 | 15 |

## Control tasks

Table S4: Descriptive statistics for the control tasks as a function of Group and Day.

| **Test** | **Component** | **Group** | | | |
| --- | --- | --- | --- | --- | --- |
|  |  | **tRNS (*M*±*SD*)** | | **Sham (*M*±*SD*)** | |
|  |  | **Day 1** | **Day 5** | **Day 1** | **Day 5** |
| *ANT* | Alerting | 23.06±15.72 | 28.19±16.24 | 24.82±23.56 | 28.72±10.69 |
|  | Orienting | 46.62±31.08 | 47.2±19.86 | 49.71±25.84 | 53.89±22.16 |
|  | Executive function | 72.38±13.08 | 64.39±12.33 | 79.8±14.65 | 69.95±17.84 |
| *Digit span* | Forward | 11.31±2.70 | 12.13±3.22 | 10.81±2.51 | 11±2.22 |
|  | Backward | 9.06±3.30 | 8.75±3.71 | 7.56±2.68 | 7.19±2.66 |
| *WIAT* | Numerical Operations | 116.44±11.43 | – | 118.13±8.88 | – |
|  | Mathematical Reasoning | 116.44±9.67 | – | 116.19±9.36 | – |
|  | Composite | 120.88±13.37 | – | 121.25±10.87 | – |

## Modelling of Calculation learning curves

A Group × Difficulty ANOVA on *B* (initial performance) revealed no effects (all *p>.1*). However, in order to control for inter-subject variability in this parameter, we used *B* as a covariate in an analysis of covariance (ANCOVA) on *α* (Snowball et al., 2013). There were no significant main effects (all *p>.1*) but a significant Group × Difficulty interaction (*F*(1, 27)=4.60, *p*<.05, η_p_^2^=.15) (see Figure S1). Subsidiary ANOVAs revealed that *α* decreased with increased difficulty in the sham group (*F*(1,11)=14.72, *p*<.001, η_p_^2^=.32), but not in the tRNS group (*p>.1*).

Figure S1: Calculation learning rate (*α*) as a function of Difficulty and Group. Error bars represent 95% confidence intervals. ***: *p*<.001

# Supplementary References

Ambrus, G. G., Paulus, W., & Antal, A. (2010). Cutaneous perception thresholds of electrical stimulation methods: Comparison of tDCS and tRNS. *Clinical Neurophysiology*, *121*(11), 1908–1914. http://doi.org/10.1016/j.clinph.2010.04.020

Fertonani, A., Pirulli, C., & Miniussi, C. (2011). Random Noise Stimulation Improves Neuroplasticity in Perceptual Learning. *The Journal of Neuroscience*, *31*(43), 15416–15423.

Iuculano, T., & Cohen Kadosh, R. (2013). The Mental Cost of Cognitive Enhancement. *The Journal of Neuroscience*, *33*(10), 4482–4486. http://doi.org/10.1523/JNEUROSCI.4927-12.2013

Newell, A., & Rosenbloom, P. S. (1981). Mechanisms of skill acquisition and the law of practice. In *Cognitive skills and their acquisition*.

Prichard, G., Weiller, C., Fritsch, B., & Reis, J. (2014). Effects of Different Electrical Brain Stimulation Protocols on Subcomponents of Motor Skill Learning. *Brain Stimulation*. http://doi.org/10.1016/j.brs.2014.04.005

Rickard, T. C. (1997). Bending the power law: A CMPL theory of strategy shifts and the automatization of cognitive skills. *Journal of Experimental Psychology: General*, *126*(3), 288–311. http://doi.org/10.1037/0096-3445.126.3.288

Snowball, A., Tachtsidis, I., Popescu, T., Thompson, J., Delazer, M., Zamarian, L., … Cohen Kadosh, R. (2013). Long-Term Enhancement of Brain Function and Cognition Using Cognitive Training and Brain Stimulation. *Current Biology*. http://doi.org/10.1016/j.cub.2013.04.045
